# DNA Methylation of TGFβ Target Genes: Epigenetic Control of TGFβ Functional Duality in Liver Cancer

**DOI:** 10.3390/cells10092207

**Published:** 2021-08-26

**Authors:** Kevin Bévant, Matthis Desoteux, Abdel Hady A. Abdel Wahab, Sabrin A. Abdel Wahab, Ayman Mohamed Metwally, Cédric Coulouarn

**Affiliations:** 1 Centre de Lutte Contre le Cancer Eugène Marquis, Inserm, University of Rennes 1, UMR_S 1242, COSS (Chemistry, Oncogenesis Stress Signaling), 35042 Rennes, France; bevantkevin@gmail.com (K.B.); matthis.desoteux@univ-rennes1.fr (M.D.); 2 Department of Cancer Biology, National Cancer Institute, Cairo University, Cairo 11796, Egypt; abdelhadynci@gmail.com; 3 Medical Laboratory Department, Students Hospital, Cairo University, Cairo 11796, Egypt; drsabrin2007@gmail.com; 4 Medical Laboratory Technology Department, College of Applied Health Science Technology, Misr University for Science and Technology (MUST), Al-Motamayez District, 6th of October P.O. Box 77, Egypt

**Keywords:** hepatocellular carcinoma, TGFβ, EMT, epigenetics, DNA methylation

## Abstract

Transforming growth factor beta (TGFβ) plays a key role in liver carcinogenesis. However, its action is complex, since TGFβ exhibits tumor-suppressive or oncogenic properties, depending on the tumor stage. At an early stage TGFβ exhibits cytostatic features, but at a later stage it promotes cell growth and metastasis, as a potent inducer of epithelial to mesenchymal transition (EMT). Here, we evaluated DNA methylation as a possible molecular mechanism switching TGFβ activity toward tumor progression in hepatocellular carcinoma (HCC). We report that decitabine, a demethylating agent already used in the clinic for the treatment of several cancers, greatly impairs the transcriptional response of SNU449 HCC cells to TGFβ. Importantly, decitabine was shown to induce the expression of EMT-related transcription factors (e.g., *SNAI1/2*, *ZEB1/2*). We also report that the promoter of *SNAI1* was hypomethylated in poor-prognosis human HCC, i.e., associated with high grade, high AFP level, metastasis and recurrence. Altogether, the data highlight an epigenetic control of several effectors of the TGFβ pathway in human HCC possibly involved in switching its action toward EMT and tumor progression. Thus, we conclude that epidrugs should be carefully evaluated for the treatment of HCC, as they may activate tumor promoting pathways.

## 1. Introduction

Liver cancer is the fifth most common cancer worldwide and the third most common cause of cancer related death [1]. Hepatocellular carcinoma (HCC) accounts for about 85% of liver primary tumors, with approximately 626,000 cases diagnosed and 598,000 deaths each year, worldwide [1]. HCC incidence has increased dramatically over the last 20 years, including in high-incidence countries [1]. Most HCC cases (about 80%) occur in sub-Saharan Africa and in Eastern Asia. In France, the incidence of HCC has increased in the last 20 years. Nonalcoholic fatty liver diseases represent the fastest growing cause of HCC, not only in France, but also in the USA and the UK [2]. In Egypt, HCC is the second most frequent cause of cancer incidence and mortality in men [1]. Recent investigations have shown the increasing importance of HCV infection in the etiology of HCC, now estimated to account for >50% of HCC cases. As an example, 69% of a cohort of 1328 patients were reported as HCV positive HCC [3]. Thus, Egypt exhibits the highest prevalence of HCV worldwide, and has experienced a dramatic rise in HCC rates [4]. In contrast to the overall mortality rate, which has declined for most cancer types, liver cancer shows the fastest increase in mortality rate [1]. Although significant progress has been made in the management of patients, HCC treatment represents an important clinical challenge [5]. When possible and applied at an early stage, surgery, including tumor resection and liver transplantation, remains the best curative option [6]. Unfortunately, HCC is usually diagnosed at an advanced stage for most patients, thus limiting the therapeutic options [6]. The efficacy of systemic therapies at advanced stages is challenged by the drug-resistant and heterogeneous nature of liver tumors. In addition, common driver mutations (e.g., *P53* and *CTNNB1*) in HCC are not currently drug-treatable [7]. Over the last two decades, functional genomics have allowed a better understanding of the mechanisms underlying HCC carcinogenesis and have unraveled the molecular heterogeneity of HCC tumors [8,9]. Thus, clinically relevant HCC subtypes have been reported [8,10,11]. Notably, we reported good- and poor-prognosis HCC subtypes associated with the activation of the transforming growth factor beta (TGFβ) pathway [11].

TGFβ is a pleiotropic cytokine from the tumor microenvironment that controls fundamental processes, including cell proliferation, apoptosis, differentiation, migration and immunity [12]. Not surprisingly, the TGFβ pathway is frequently deregulated in cancer, including liver cancers. TGFβ plays an important role in liver carcinogenesis by contributing to all stages of tumor onset and progression [13,14,15]. Thus, TGFβ represents a promising candidate for the development of innovative therapeutic strategies [16]. However, targeting the TGFβ pathway in cancer is complex, given that TGFβ exhibits either tumor-suppressive or oncogenic properties, depending on the tumor stage. At an early stage, TGFβ acts as a powerful cytostatic factor on pre-malignant cells, but at a later stage TGFβ promotes cell growth and favors metastasis of tumor cells, notably as a potent inducer of epithelial to mesenchymal transition (EMT) [17]. So far, the molecular mechanisms underlying the functional duality of TGFβ during tumor progression are not fully understood. Several positive and negative regulators (e.g., SARA, SMAD7, SKIL), of which the expression is context dependent, have been shown to tightly regulate the TGFβ pathway [18]. Thus, modulating the expression or the activity of these regulators and/or effectors associated with tumor-suppressive versus tumor-promoting features of the TGFβ pathway, may greatly impact the course of tumor progression.

Epigenetic mechanisms, such as chromatin remodeling and DNA methylation, modulate cancer-associated processes (e.g., cell proliferation, invasion, metastasis), and thus HCC onset and progression [19]. These mechanisms are involved in the fine-tuning of gene expression. DNA methylation is catalyzed by DNA methyltransferases (DNMTs), a family of enzymes including DNMT1, DNMT3A, and DNMT3B. DNMTs place a methyl group next to guanosine on CpG dinucleotides, which frequently build clusters in the promoter regions of genes [20]. Herein, we evaluated DNA methylation as a possible mechanism regulating the activity of the TGFβ pathway in HCC. We hypothesized that shifting TGFβ signaling from tumor-suppressive toward pro-metastatic activities may be directly influenced by the degree of methylation of genes involved in the TGFβ pathway. To test this hypothesis, DNA methylation of TGFβ regulators and effectors was analyzed in vitro in HCC cells, and further evaluated on resected human HCC samples to determine the clinical relevance of DNA methylation.

## 2. Materials and Methods

### 2.1. Human HCC Samples

Human HCC and non-tumor tissues were isolated from patients undergoing surgery as a primary therapeutic modality during the period from 2001 to 2003 at the National Cancer Institute, Cairo University, Egypt. Department and Institutional approval was obtained. HCC diagnosis was confirmed by histopathological examination of the resected tissues by 2 independent pathologists. Clinical data as well as follow-up studies of the patients were retrospectively collected (n = 16 patients). Patients’ consent was obtained before sample collection. The study was conducted according to the Declaration of Helsinki.

### 2.2. Culture of HCC Cells

HCC cell lines were purchased from ATCC (www.lgcstandards-atcc.org accessed on 12 April 2015) and cultured as previously described [10]. ATCC performed cell lines authentication by STR DNA profiling. Cells were treated with 1 ng/mL recombinant human TGFβ1 (R&D system, Minneapolis, MN, USA) and 100µM 5-azacytidine (decitabine) (Sigma-Aldrich, St. Louis, MO, USA), alone or in combination. Briefly, overnight serum starvation was applied to all cell cultures. Cells were subsequently incubated in the presence of decitabine (versus control) for 8 h. After 8 h, cells were incubated for 16 h with TGFβ (versus control) in the presence or absence of decitabine (Supplementary Figure S1).

### 2.3. Gene Expression Profiling

Total RNA was extracted using a miRNAeasy kit (Qiagen, Courtabeuf, France) according to the manufacturer’s instructions. Gene expression profiling was performed using a low-input QuickAmp labeling kit and human SurePrint G3 8 × 60 K microarrays (Agilent Technologies, Santa Clara, CA, USA). Differentially expressed genes were identified by a two-sample univariate *t* test and a random variance model, as previously described [21]. RT-qPCR was performed using a SYBR Green (Applied Biosystems, Carlsbad, CA, USA) and analyzed as previously described [21].

### 2.4. DNA Extraction

DNA was extracted from 5–25 mg HCC tissues and adjacent non-tumor human tissues using a QIAamp Fast DNA Tissue Kit (Qiagen, cat. no. 51404). For each sample the following cocktail was added: 200 μL AVE, 40 μL VXL, 1 μL DX Reagent, 20 μL Proteinase K and 4 μL RNase A (100 mg/mL). Tissue samples were homogenized by vortexing for 5 min, and were then incubated in a thermomixer at 1000 rpm for 10 min at 56 °C, prior to adding 265 μL MVL Buffer. The mixture was added to the QIAamp Mini spin column and processed according to the manufacturer’s instructions. DNA concentration and purity were measured using a Nanodrop.

### 2.5. Promoter Methylation Analysis

Promoter methylation for *TRFBR2* (EPHS110111-1A), *SMAD4* (EPHS106631-1A), *SMAD7* (EPHS106615-1A) and *SNAI1* (EPHS109344-1A) was studied using the Methyl Screen technology by EpiTect Methyl II Primer Assay kits (Qiagen, cat. 335002). The restriction digestions were performed using the EpiTect Methyl II DNA Restriction Kit (Qiagen, cat. 335452). Amplification was performed using an Applied Biosystem VIIA7 thermocycler. Analysis was performed using the dedicated EpiTect Methyl II PCR Array Microsoft Excel template (www.sabiosciences.com/dna_methylation_data_analysis.php accessed on 20 November 2020). Briefly, CT values were exported to the data analysis sheet and the results (percentage of promoter methylation for each gene) were automatically generated.

## 3. Results

### 3.1. Decitabine Impairs the Transcriptional Response of SNU449 HCC Cells to TGFβ

**Figure 1:**
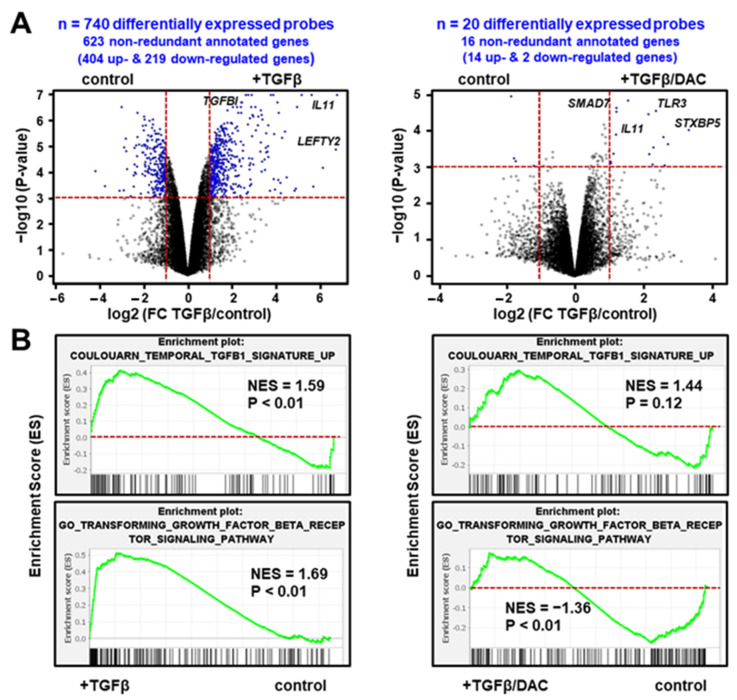
Decitabine impairs the transcriptional response of SNU449 HCC cells to TGFβ. (**A**) Volcano plot highlighting differentially expressed probes (blue dots) in SNU449 cells treated with TGFβ (16 h) in the absence (left panel) or presence (right panel) of decitabine (DAC). Probes were selected based on the significance of the differential expression in the experimental conditions (horizontal red line; *p* < 0.001) and the level of induction or repression (vertical red lines; fold change >2). In total, 740 probes were differentially expressed upon TGFβ treatment in the absence of DAC, but only 20 probes in the presence of DAC, using the same selection criteria. (**B**) Gene set enrichment analysis (GSEA) using the gene expression profiles of SNU449 cells treated with TGFβ in the absence (left panels) or presence (right panels) of decitabine. Shown are the enrichment plots of two curated TGFβ-associated gene expression signatures. NES: Normalized Enrichment Score.

Gene expression profiling was performed in the SNU449 HCC cell line treated with TGFβ (16 h) in the absence or presence of decitabine. As shown in Figure 1A (left panel), 740 probes, corresponding to 623 well-annotated non-redundant genes, were differentially expressed by TGFβ (i.e., with a fold-change TGFβ/control FC > 2 and a *p*-value < 0.001). Validating the gene selection, the highlighted gene signature included well-known TGFβ targets, including upregulated genes (e.g., *COL4A4*, *IL11*, *LEFTY2*, *SERPINE1*, *SMAD7*, *SNAI1* or *TGFBI*) and down-regulated genes (e.g., *AQP1*, *RAB17*, *SORBS2*). Accordingly, Gene Set Enrichment Analysis (GSEA) demonstrated that an experimentally well-defined TGFβ signature, which we established previously using *Tgfbr2* knockout mice [11], was significantly enriched (NES = 1.59; *p* < 0.01) in the gene expression profiles of SNU449 cells exposed to TGFβ (Figure 1B, upper left panel). An independent curated TGFβ signature entitled “GO_Transforming_Growth_Factor_Beta_Receptor_Signaling_Pathway” from the Gene Ontology Consortium was similarly enriched (Figure 1B, lower left panel). The expression of 5 TGFβ-responsive genes (*IL11*, *SERPINE1*, *SMAD7*, *SNAI1* and *TGFBI*) was further validated in other liver cancer cell lines (Supplementary Figure S2A).

**Figure 2:**
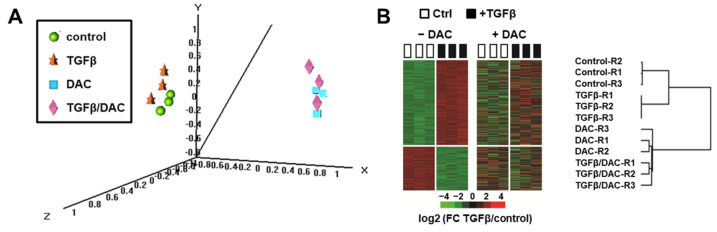
Clustering analysis of gene expression profiles of SNU449 cells upon TGFβ and decitabine (DAC) treatments. (**A**) Multidimensional scale (MDS) plot of samples colored by experimental groups, based on the expression of 28,101 probes (global gene expression profiles). (**B**) Hierarchical clustering analysis based on the expression of 740 probes differentially expressed in SNU449 cells upon TGFβ treatment (*p* < 0.001 and fold-change FC > 2) in the absence of decitabine (−DAC, left heatmap). The right heatmap and the dendrogram demonstrate that DAC co-treatment abolished the modulation of TGFβ responsive genes (R1–3: Replicates 1–3).

Importantly, in the presence of decitabine, only 20 probes, corresponding to 16 well-annotated non-redundant genes, were differentially expressed by TGFβ using the same statistical criteria (i.e., with a TGFβ/control FC > 2 and a *p*-value < 0.001). Among these genes, *SMAD7* and *IL11* remain upregulated by TGFβ (Figure 1A, right panel). As shown in Figure 1B (right panels), the curated TGFβ signatures were not enriched anymore in the gene expression profiles of SNU449 cells exposed to TGFβ and decitabine. In fact, a statistically significant negative enrichment (NES = −1.36; *p* < 0.01) was observed with the TGFβ signature established by the Gene Ontology Consortium. In addition, principal component analysis using the whole gene expression profiles (Figure 2A) and clustering analysis based on the expression of 740 probes differentially expressed upon TGFβ exposure (Figure 2B), demonstrated that the transcriptomic profile of SNU449 cells exposed to TGFβ in the presence of decitabine (TGFβ/DAC) was closer to the transcriptomic profile of cells exposed to decitabine (DAC) than to TGFβ or controls. Altogether, these data demonstrate that decitabine greatly impairs the transcriptional response of SNU449 HCC cells to TGFβ.

### 3.2. Decitabine Inhibits the Expression of Members of Canonical SMAD/TGFβ Signaling Pathway

**Figure 3:**
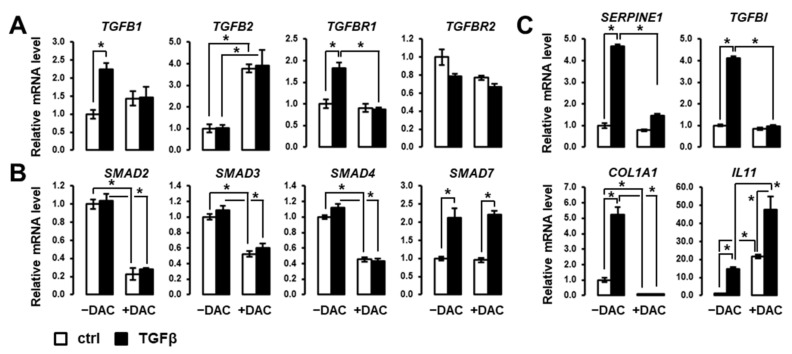
Decitabine greatly impacts the expression of regulators and effectors of the canonical SMAD/TGFβ signaling pathway. Analysis of the expression of genes encoding (**A**) TGFβ ligands and receptors, (**B**) SMADs transducers and regulators of TGFβ signaling, and (**C**) well-known TGFβ targets, in SNU449 cells treated with TGFβ (black bars) versus control (white bars) in the absence (left 2 bars) or presence (right 2 bars) of decitabine (DAC). SNU449 HCC cells were treated alone or in combination with 1 ng/mL TGFβ1 and 100µM decitabine (DAC) for 24 hrs. * *p* < 0.05 (± TGFβ, ± DAC).

Next, we focused on the impact of decitabine on the expression of genes involved in the canonical TGFβ pathway, including TGFβ ligands, receptors, intracellular signal transducers and key target genes. As shown in Figure 3A, decitabine inhibits the induction by TGFβ of *TGFB1* and *TGFBR1*. Decitabine induces the expression of *TGFB2* but has no impact on the expression of *TGFBR2*. Decitabine drastically inhibits the expression of *SMAD2-4,* which is critical to transducing the TGFβ signal from the membrane to the nucleus (Figure 3B). The expression of *SMAD7*, acting as a TGFβ-induced negative feedback regulator of the signaling pathway, is not affected by decitabine (Figure 3B). Accordingly, key target genes of the canonical SMAD-dependent TGFβ signaling pathway (e.g., *SERPINE1*, *TGFB1*, *COL1A1*) are repressed in the presence of decitabine (Figure 3C). Interestingly, the induction by TGFβ of *IL11*, a cytokine of the IL6/GP130 family involved in multiple cancer hallmarks, including cell migration and invasion [22,23], is greatly enhanced by decitabine (Figure 3C). These data suggest that decitabine suppresses the expression of members of canonical SMAD/TGFβ signaling pathway and may switch the actions of TGFβ toward pro-metastatic features.

### 3.3. Decitabine Induces the Expression of EMT-Related Transcription Factors in SNU449 Cells

**Figure 4:**
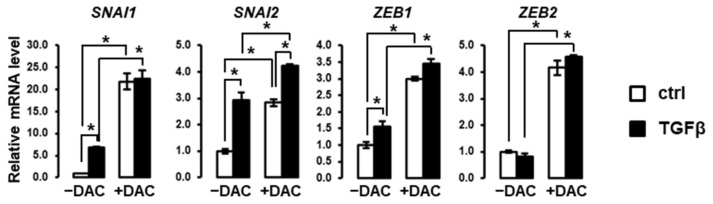
Decitabine enhances the expression of EMT-associated transcription factors. Expression analysis was performed in SNU449 cells treated with TGFβ (black bars) versus control (white bars) in the absence (left 2 bars) or presence (right 2 bars) of decitabine (DAC). SNU449 cells were treated alone or in combination with 1 ng/mL TGFβ1 and 100µM decitabine (DAC) for 24 hrs. * *p* < 0.05.

Based on the above observations, we evaluated the expression of key transcriptional regulators of EMT in SNU449 cells treated with TGFβ in the absence or presence of decitabine. As shown in Figure 4, decitabine greatly enhances the expression of EMT-associated transcription factors, including *SNAI1*, *SNAI2*, *ZEB1* and *ZEB2*. Notably, decitabine enhanced the induction of these transcription factors by TGFβ. These data suggest that these EMT-related transcription factors are repressed by methylation in SNU449 cells.

The results were further validated by RT-qPCR in Huh6 and Hep3B HCC cell lines (Supplementary Figure S2B). In addition, we have performed a meta-analysis of a gene expression dataset, which reported the impact of 5-azacitidine on several HCC cell lines (GSE112788). As shown in Supplementary Figure S2C, *SNAI1*, *SNAI2* and *ZEB1* as well as *IL11* were induced only in some cell lines (particularly HLE and HLF). Very interestingly, we previously reported that these specific cell lines, similar to SNU449, were associated with the so-called late pro-metastatic TGFβ signature [11]. These data support the idea that epidrugs could be detrimental in specific HCC tumors by activating genes encoding EMT-associated transcription factors.

### 3.4. Promoter Methylation of TGFβ-Associated Genes in SNU449 HCC Cells

**Figure 5:**
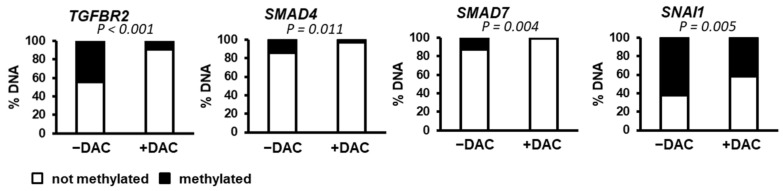
Methylation status of the promoters of genes associated with the TGFβ pathway. DNA methylation of TGFBR2, SMAD4, SMAD7 and SNAI1 promoters was evaluated in SNU449 HCC cells in the absence (left bar, −DAC) or presence of decitabine (right bar, +DAC). *p* value was determined by using a chi-square test (Yates value corrected for continuity).

As shown in Figure 3 and Figure 4, the expression of several genes linked to the TGFβ signaling pathway was modulated in the presence of decitabine. Thus, we directly evaluated the degree of promoter methylation of key candidates in SNU449 HCC cells in the absence or presence of decitabine. The data demonstrated that the promoter of *SNAI1* is highly methylated (>60%) in SNU449 HCC cells (Figure 5). Accordingly, decitabine significantly reduces the percentage of methylated DNA (Figure 5), in agreement with the previously observed induced expression (Figure 4). A similar observation was made for *TGFBR2* (Figure 5), although no significant change in expression was observed in the presence of decitabine (Figure 3). The promoters of *SMAD4* and *SMAD7* were methylated at low levels (<20%) in SNU449 HCC cells (Figure 5).

### 3.5. Clinical Relevance of Promoter Methylation of TGFβ-Associated Genes in Human HCC

Next, we evaluated the specific promoter methylation of the four genes from the TGFβ signaling pathway (*TGFBR2*, *SMAD4*, *SMAD7* and *SNAI1*) evaluated in SNU449 cells in resected human HCC tumors, as well as in the surrounding non-tumor tissues. The median methylation of promoters in HCC was 58% for *TGFBR2* (Range 99.91, min. 0.09, max. 100), 2% for *SMAD4* (Range 99.88, min. 0.12, max. 100), 50% for *SMAD7* (Range 99.99, min. 0.01, max.100) and 40% for *SNAI1* (Range 100, min. 0, max. 100). In the surrounding non-tumor tissues (NT), the median methylation of promoters was 75% for *TGFBR2* (Range 99.68, min. 0.32, max. 100), 51% for *SMAD4* (Range 47.66, min. 26.78, max. 74.44), 23% for *SMAD7* (Range 99.85, min. 0.05, max. 99.9) and 50% for *SNAI1* (Range 99.17, min. 0.57, max. 99.74). No statistically significant difference in gene methylation was observed between NT and HCC groups. Indeed, an important variation in the methylated/unmethylated DNA ratio was observed in human biological samples (Supplementary Figure S3), suggesting that specific methylation profiles could be associated with clinically relevant HCC subtypes. To test this hypothesis, the clinical relevance of promoter methylation of *TGFBR2*, *SMAD4*, *SMAD7* and *SNAI1* genes in HCC was evaluated by correlating the percentage of methylation/non-methylation with clinical and biological parameters, including overall survival, tumor grade, recurrence, metastasis and serum alpha-fetoprotein levels. The median overall survival of the studied cases was 14.1 months (Range 31.5, min. 0.13, max. 31.63) while the median disease-free survival was 9.5 months (Range 16.1, min. 0.33, max. 16.43). No significant correlation was observed between promoter methylation of genes studied and survival (data not shown).

**Figure 6:**
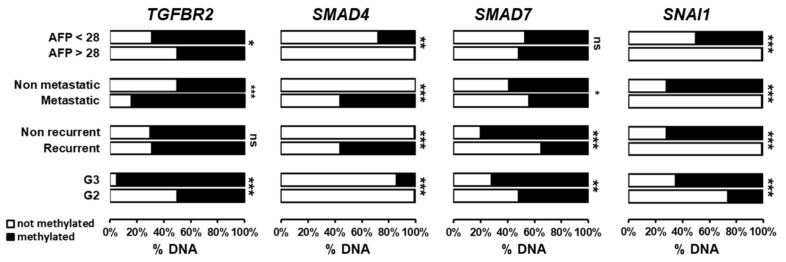
Methylation status of the promoter of *TGFBR2*, *SMAD4*, *SMAD7* and *SNAI1* genes in human HCC tumors and correlation with clinical and biological parameters. The percentage of DNA methylation was correlated with tumor grade (G2 vs. G3), recurrence, metastasis and serum alfa-fetoprotein levels. *** *p* < 0.001; ** *p* < 0.01; * *p* < 0.05; ns *p* > 0.05.

However, as shown in Figure 6, *TGFBR2* promoter hypermethylation in HCC is significantly associated with high tumor grade and metastasis (*p* < 0.001) and to a lesser extend with lower alfa fetoprotein level (*p* = 0.029) but no significant correlation is found with recurrence. Higher *SMAD4* promoter methylation is significantly associated with high tumor grade, recurrence, metastasis, and elevated AFP (*p* < 0.01). Interestingly, low promoter methylation of *SMAD7* was associated with lower tumor grade but high recurrence rate (*p* < 0.001) and metastasis (*p* = 0.05). No significant correlation was found with AFP level (Figure 6). *SNAI1* promoter methylation profiles were significantly correlated with all parameters. Thus, HCC associated with metastasis and recurrence exhibited significantly lower *SNAI1* promoter methylation, reflecting a higher expression. Demethylation of *SNAI1* promoter is correlated with elevated AFP levels (*p* < 0.001).

## 4. Discussion

Carcinogenesis results in the accumulation of genetic and epigenetic alterations. Previous studies have highlighted the role of DNA methylation in the early steps of carcinogenesis, in particular its contribution to chromosomal instability [24]. Over the past two decades, the genomic landscape of these alterations, including mutation spectrum affecting oncogene-driven signaling pathways, has been highlighted in several cancers, including HCC [25]. Epigenetic alterations have been also shown to contribute to HCC [19]. Supporting a critical epigenetic control of HCC onset and progression, it was recently reported that alterations in DNA methylation occurred in the early pre-neoplastic phases of HCC development and contributed to the deregulation of cancer related genes and pathways [26]. It was also reported that DNA methylation patterns in cirrhotic or fibrotic liver tissues are clinically relevant to identifying those patients at risk of HCC development and de novo recurrence after surgery [26]. We previously reported clinically relevant gene expression signatures in human HCC associated with the dual role of TGFβ and predicting HCC with better and poor prognosis [11]. In this study, we hypothesized that the functional duality of the TGFβ pathway in HCC (e.g., tumor-suppressive versus tumor-promoting activities) could depend, at least partly, on the degree of DNA methylation in the promoter of TGFβ-responsive genes and/or regulators of the TGFβ pathway. We tested this hypothesis in SNU449 HCC cell line and in human HCC.

First, we showed that decitabine and subsequently a global demethylation impaired the TGFβ signaling and induced the expression of several EMT master genes (e.g. *SNAI1/2*, *ZEB1/2*). Previous studies highlighted the importance of DNA methylation on cellular responses to SMADs. Martin et al. showed that TGFβ treatment induces a change in the methylome of HCC cell lines HepG2 and Huh7 mediated by DNMT3, and that hypermethylation of some core genes increases gene expression [27]. This regulation could explain, at least partially, our observation that global demethylation represses many TGFβ target genes. Global demethylation and disruption of SMAD signaling has been previously associated with a shift toward an epithelial phenotype [28]. Our results demonstrated that several members of the TGFβ pathway were subjected to epigenetic regulations in HCC. Indeed, the expression of these genes is greatly influenced by the degree of promoter methylation, as demonstrated in the SNU449 HCC cell line and in patient tumor samples. In addition, our results demonstrated that the degree of methylation in TGFβ target genes in HCC tumors was a clinically relevant factor associated with the risk of metastasis and tumor recurrence in patients.

Our results showed that the expression of *SMAD4*, a member of the canonical TGFβ signaling responsible for the transmission of the signal from the cell membrane to the nucleus [29,30], is inhibited by decitabine in the SNU449 cell line. A possible explanation is that decitabine activates, by demethylation, other gene(s) which, in turn, act as inhibitors of *SMAD4* expression. More interestingly, the results in human HCC indicated that the pattern of *SMAD4* methylation was clinically relevant. Thus, hypermethylation of *SMAD4* promoter (usually associated with gene inactivation) was predictive of HCC recurrence and metastasis. These results suggested that the epigenetic inhibition of *SMAD4* expression by DNA methylation in poor-prognosis HCC may contribute to the loss of the tumor-suppressive arm of the canonical TGFβ pathway, as is documented in regard to other tumors, including pancreatic and colorectal carcinoma [31,32]. Indeed, as a tumor suppressor gene, *SMAD4* is frequently inactivated in cancer by several mechanisms, mostly by genetic alterations [33] but also by promoter methylation. Thus, although mutations in *SMAD4* are rare events in prostate cancer and HCC, methylation of its promotor is commonly detected and associated with reduced expression [34]. It is important to emphasize that HCC samples analyzed in our study derived from patients at early stages, allowing tumor removal by surgery. Accordingly, *SMAD4* promoter was mostly unmethylated (i.e., active) and possibly exerting its tumor suppressor role to prevent recurrence and metastasis in these patients [35,36]. This observation is in agreement with the tumor suppressive role of the canonical SMAD-dependent TGFβ pathway in the early stages of HCC [14,16,37]. However, a dual function of SMAD4 during the course of HCC onset and progression, where it may exert a tumor-suppressive function at an early stage and an oncogenic function at later stages, should be taken into consideration. Indeed, while the tumor-suppressive functions of SMAD4 are well established in mediating cell cycle arrest and apoptosis, it is clear that SMAD4 is also required for tumor progression, notably by mediating TGFβ-induced expression of EMT transcription factors, such as *SNAIL*, *SLUG*, *TWIST* and *ZEB* [33,38,39,40]. In the liver, it was shown that SMAD4 knockdown significantly reduced formation and growth of tumors through SNAIL upregulation, suggesting that TGFβ promotes EMT in a SMAD4-dependent manner [33,41]. Thus, one can expect that the functional duality of the TGFβ pathway is supported in part by the functional duality of its regulators and effectors.

In regard to *SMAD7*, which is a negative feedback regulator for the TGFβ pathway, although its expression was not affected by decitabine in SNU449 cells, we observed that its promoter was hypomethylated (i.e., signaling a possible higher expression/activity) in early stage human HCC, especially in poor-prognosis tumors associated with tumor recurrence. Wang et al. reported similar results where *SMAD7* was upregulated in human HCC samples with poor prognosis [42]. SMAD7 was shown to contribute to liver carcinogenesis by activating the YAP/NOTCH signaling cascade and inducing an EMT signature [42]. Park et al. also documented an increased SMAD7 immunoreactivity in advanced, but not in early, HCC [43]. However, conflicting results have been also reported, where a decreased expression of *SMAD7* in HCC patients was associated with early recurrence and poor prognosis [44]. In the later study, it seems that the decreased expression of *SMAD7* resulted from an indirect effect, notably through the overexpression of miR-216a/217 [44]. The significance of *TGFBR2* promoter methylation in our study was not as high as the other markers. A significant association was observed with tumor grade and metastasis but not with recurrence. The association between *TGFBR2* low expression and high tumor grade tumor was reported in other studies [45,46]. In-depth functional studies will be needed to decipher the correlation between *TGFBR2* and the other TGFβ target genes.

Moreover, our data clearly demonstrated that the expression of EMT-associated transcription factors from the SNAI and ZEB families was greatly enhanced by decitabine in SNU449 cells, suggesting that these genes could be inactivated by promoter methylation. It is possible that the induction of EMT master genes by decitabine is independent of TGFβ signaling, considering that most of these genes are not induced by TGFβ in the presence of decitabine. Interestingly, data from the literature indicate that silencing of DNMTs is associated with the induction of EMT and stemness in several cell types, including prostate cancer [47], bladder cancer [48], breast cancer [49] and liver cancer [50]. Altogether these data support our hypothesis that reduced activity of DNMTs (either by silencing or pharmacological inhibition) may result in the activation of EMT-associated genes or other tumor-promoting genes. Based on the design of our experiments (i.e., short term cell cultures), no obvious signs of aggressiveness were observed in HCC cells upon simultaneous treatment with decitabine and TGFβ. However, it is conceivable that long term experiments may result in a full EMT phenotype, possibly associated with stemness and drug resistance features. Indeed, data from the literature provide evidence for the significance of epigenetic mechanisms regulating liver cancer stem cells (CSCs), particularly in a context using inhibitors of DNA methyltransferases. Thus, it was demonstrated in human HCC cell lines that inhibition of DNA methylation by zebularine, an inhibitor of DNMT1, resulted in an increase in highly tumorigenic cells within the Side Population (SP) fraction, which exhibited features of CSCs and a gene expression profile predicting a poor clinical outcome (reduced survival and early recurrence) in patients with HCC [51,52]. Accordingly, in human HCC, we observed that a low *SNAI1* promoter methylation is a risk factor for tumor recurrence and metastasis. These results underscore demethylation in early stages of liver cancer as a risk factor for tumor progression [53,54,55]. They also provide critical information that may help to develop a strategy for the treatment of HCC patients according to the epigenetic pattern of genes associated with the TGFβ pathway.

Epigenetic modifications are able to alter the expression of genes without disturbing the genomic sequence, which makes them promising targets for drugs in cancer treatment [56]. The first FDA-approved epi-drugs were HDAC and DNMT inhibitors for the treatment of hematological malignancies [56,57]. Interestingly, these drugs are now extensively tested in solid tumors, including HCC [58,59,60]. The effects of DNMT inhibitors on HCC cells have been widely explored. In response to these drugs, tumor suppressor genes are expressed and HCC cells partially retro-differentiated with a higher sensitivity to sorafenib [61]. Thus, current evidence suggests that epi-drugs are promising therapies for HCC patients. Epigenetic modulation has been also recently reported as a novel potential strategy to boost immunotherapy in HCC by stimulating T cell trafficking into tumor microenvironment [62]. However, because of their pleiotropic effects, it is noteworthy to consider the putative adverse effects of these drugs when administered to patients, notably in regard to the modulation of specific cell signaling pathways, as exemplified for the TGFβ pathway in our study. Indeed, our study highlights the complex influence of epigenetic mechanisms, especially promoter methylation, on the regulation of the TGFβ pathway, and the consequent complexity of targeting this pathway in cancer. Thus, decitabine may not be the treatment of choice for early-stage HCC, as it may switch the actions of TGFβ toward pro-metastatic features. This may occur though indirect inhibition of *SMAD4* and activation of both *SMAD7* and *SNAI1,* resulting in promotion of EMT and future recurrence and/or metastasis.

## 5. Conclusions

In conclusion, our study observed an epigenetic control of TGFβ functional duality in liver cancer, *SMAD4* demethylation being identified as a tumor-suppressive event possibly preventing tumor metastasis and recurrence in early HCC. In addition, the study suggested that *SMAD7* and *SNAI1* hypomethylation is associated with tumor recurrence. Decitabine is thought to switch the actions of TGFβ toward pro-metastatic features by indirect inhibition of *SMAD4* expression, and activation of both *SMAD7* and *SNAI1,* resulting in recurrence and metastasis. Thus, decitabine may not be the treatment of choice for early-stage HCC.

## Data Availability

The data that support the findings of this study are available from the corresponding authors, [AMM and CC], upon reasonable request.

## Supplementary Materials

The following are available online at https://www.mdpi.com/article/10.3390/cells10092207/s1, Figure S1: Summary of the experimental study design, Figure S2: Decitabine induces the expression of EMT-associated transcription factors in aggressive HCC cell lines, Figure S3: Methylation profiles of TGFβ-associated genes in human liver cancer.
