# Supplementary material for: DNA Methylation of TGFβ Target Genes: Epigenetic Control of TGFβ Functional Duality in Liver Cancer
Source: Cells. 2021 Aug 26;10(9):2207. doi: 10.3390/cells10092207 (PMC8468746; doi:10.3390/cells10092207)
Supplement: Supplementary file 1 [file cells-10-02207-s001.zip › cells-1318481-supplementary.pdf]

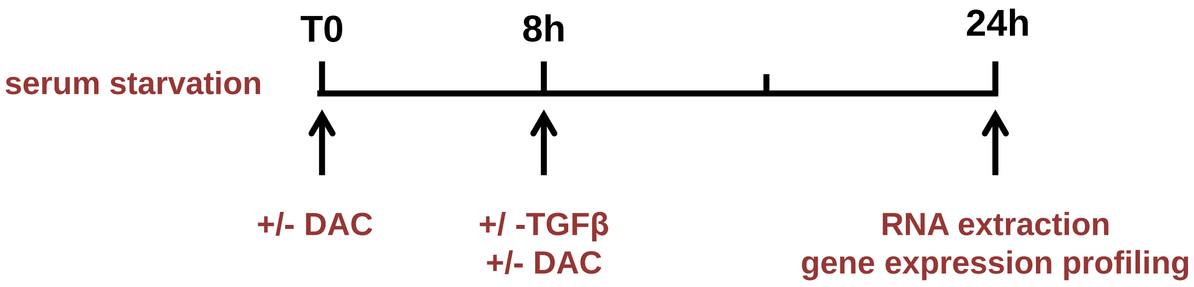

Supplementary Figure S1: Summary of the experimental study design.

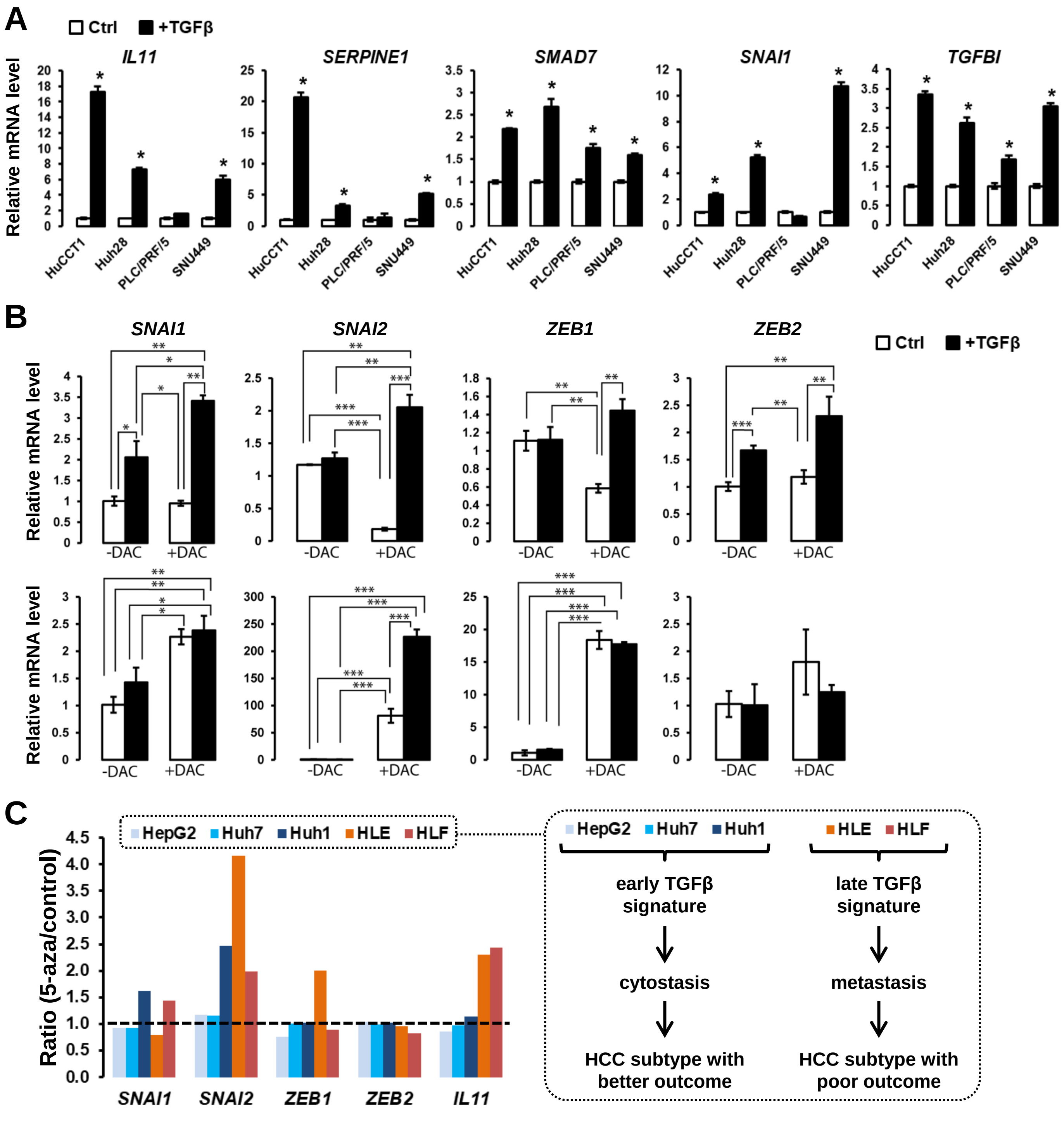

Supplementary Figure S2: Decitabine induces the expression of EMT-associated transcription factors in aggressive HCC cell lines. **(A)** RT-qPCR validation of TGFβ-responsive genes in PLC/PRF/5, HuCCT1, Huh28 and SNU449 cells (1ng/mL TGFβ; 16hrs). **(B)** RT-qPCR validation in Hep3B (upper panels) and HuH6 (lower panels) cells treated with TGFβ (black bars) versus control (white bars) in absence (left 2 bars, -DAC) or presence (right 2 bars, +DAC) of decitabine. Cells were treated alone or in combination with 1ng/mL TGFβ1 and 100µM decitabine (DAC) for 24hrs. * P<0.05, ** P<0.01, *** P<0.001 (+/-TGFβ, +/- DAC). **(C)** Meta-analysis of gene expression in HCC cells exposed to 5-azacitidine for 3 days (GSE112788). Shown are the gene expression ratios 5-azacitidine versus control. Right panel: features of HCC cells based on Coulouarn et al., *Hepatology*, 2008 [11].

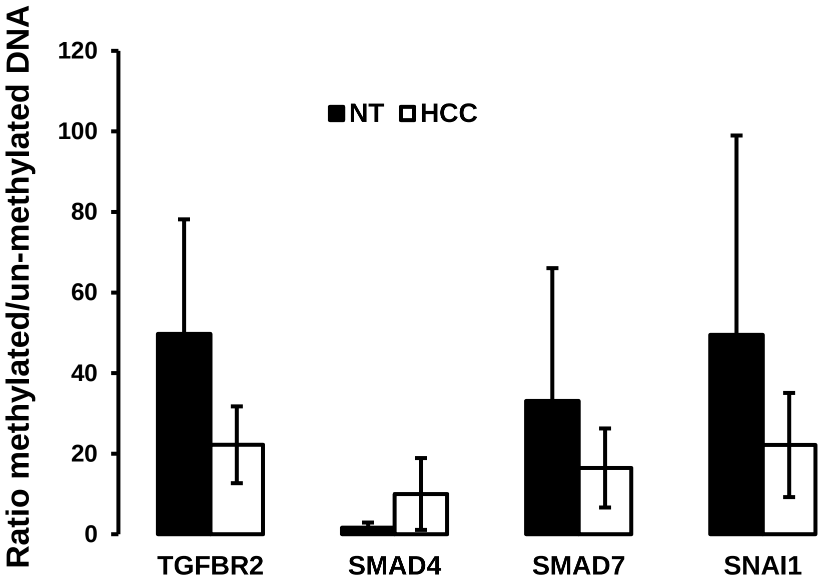

Supplementary Figure S 3: Methylation profiles of TGFβ-associated genes in human liver cancer. Ratio of methylated/un-methylated DNA in each experimental group (HCC tumors versus NT). Data are represented as mean +/- sem (P>0.05).
